# Fimbriae potentiate *Aggregatibacter actinomycetemcomitans* for periodontal disease

**DOI:** 10.1016/j.crmicr.2026.100566

**Published:** 2026-02-01

**Authors:** Yanyan Fu, Qiqi Pan, Jan Maarten van Dijl

**Affiliations:** University of Groningen, University Medical Center Groningen, Department of Medical Microbiology, Groningen, the Netherlands

**Keywords:** *Aggregatibacter actinomycetemcomitans*, Fimbriae, Virulence

## Abstract

*Aggregatibacter actinomycetemcomitans* is a major contributor to aggressive periodontitis and has been increasingly implicated in systemic conditions, such as rheumatoid arthritis and Alzheimer’s disease. Among its diverse virulence mechanisms, fimbriae have emerged as central factors driving colonization, immune evasion, and pathogenicity. This review highlights recent findings showing that fimbriae not only facilitate adhesion and biofilm formation, but also contribute to maintaining bacterial cell envelope integrity. The transition from rough (fimbriated) to smooth (non-fimbriated) colony phenotypes upon *in vitro* culturing is associated with significant changes in virulence, including increased release of outer membrane vesicles (OMVs) and extracellular cytoplasmic proteins (ECPs). These changes suggest a trade-off between surface structure expression and secretion-based virulence. Importantly, fimbriae-rich strains are clinically relevant and more virulent in host models. The combined evidence implies that fimbriae and key virulence factors, such as leukotoxin A, act cooperatively in pathogenesis, as fimbriae-mediated adhesion and persistence will enhance local toxin delivery, killing of immune cells and immune evasion. Together, these insights point to fimbriae as key targets for novel therapeutic and vaccination strategies aimed at controlling periodontal and related systemic diseases.

## Highlights

• *Aggregatibacter actinomycetemcomitans* is a causative agent of periodontitis.
• Fimbriae are key modulators of *A. actinomycetemcomitans* virulence.
• Fimbriae drive aggregation, colonization, immune evasion and pathogenicity.
• Fimbriae limit bacterial vesicle and extracellular cytoplasmic protein release.
• Fimbriae are key therapeutic targets to control periodontal disease.

## Graphical abstract

**Figure:**
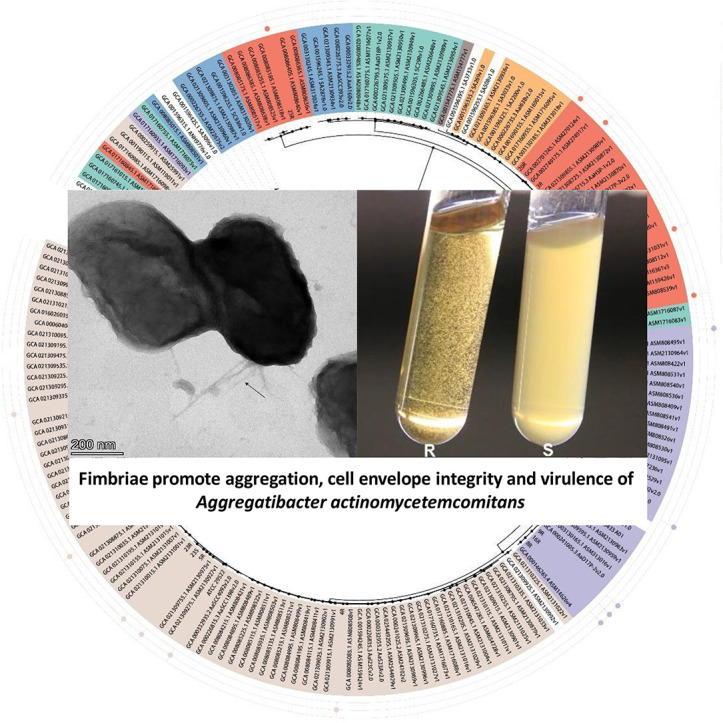

## Periodontitis – the most prevalent neglected disease

The most prevalent disease on planet earth, periodontitis, is an inflammatory disease that irreversibly destroys the soft tissues and bone surrounding our teeth (Könönen et al., 2019). Localized aggressive forms of periodontitis lead to tooth destruction, especially the permanent first molars and incisors of teenagers and young adults (Fine et al., 2019). Periodontitis has been associated with a dysbiosis of the oral microbial community that colonizes the hard surfaces of teeth and the mucosal tissues of the oral cavity (Fu et al., 2022). In healthy individuals, the oral microbiota co-evolves with the host, leading to a harmonious coexistence of the different microorganisms and the periodontal tissues (Di Stefano et al., 2022). However, once this balance is broken due to lifestyle factors, including inadequate oral hygiene, unhealthy diets and smoking, or due to the use of antibiotics, the oral microbiota will change (Santonocito et al., 2022). The resulting oral dysbiosis creates a favorable environment for oral pathogens, such as *Porphyromonas gingivalis* and *Aggregatibacter actinomycetemcomitans*, which will trigger further changes of the microbial communities and lead to periodontal inflammation and destruction (Di Stefano et al., 2022; Xu et al., 2025). Such pathogens may even impact on the behavior of other pathogens that are unrelated to periodontitis, as was recently reported for the major human pathogen *Staphylococcus aureus*, which was shown to aggregate due to the binding of outer membrane vesicles (OMVs) excreted by *P. gingivalis* (du Teil Espina et al., 2022). As bacterial aggregation is a first step towards biofilm formation, the latter observation underscores the view that an increasing abundance of oral pathogens like *P. gingivalis* can have serious repercussions for infection of the oral cavity and beyond (du Teil Espina et al., 2022). Of note, *A. actinomycetemcomitans* is a facultative anaerobe, in contrast to many other major periodontal pathogens, such as *P. gingivalis,* that are obligate anaerobes. This metabolic flexibility gives *A. actinomycetemcomitans* the possibility to colonize oxygenated niches in the periodontal pocket, allowing early biofilm development. Consequently, *A. actinomycetemcomitans* will have a competitive advantage, as obligate anaerobic periopathogens preferentially establish themselves later within oxygen-depleted environments of the periodontal pocket.

Importantly, there are significant differences in the composition of the oral microbiota in individuals with mild or severe periodontitis (Plachokova et al., 2021). This reflects the fact that some bacteria have a higher propensity to cause inflammation than other bacteria. Accordingly, the outgrowth of some notorious oral pathogens, like *P. gingivalis* and *A. actinomycetemcomitans*, is strongly associated with systemic inflammation even though their abundance may remain relatively low (Cortelli et al., 2020; Fine et al., 2020).

## *A. actinomycetemcomitans* – an evolving pathogen

*A. actinomycetemcomitans* has attracted attention, because it is associated with aggressive forms of periodontitis in young adults (Nørskov-Lauritsen et al., 2019). In general, the preferred ecological niche of *A. actinomycetemcomitans* is the oral cavity (van Winkelhoff et al., 1993). However, it may translocate across the epithelial barrier, causing dangerous infections of the blood, heart valves, joints, soft tissues, bone and brain (Nedergaard et al., 2019; Sharma et al., 2017; Rahamat-Langendoen et al., 2011). Moreover, *A. actinomycetemcomitans* has also been implicated in severely debilitating systemic diseases, such as the autoimmune disorder rheumatoid arthritis (RA) and Alzheimer’s diseases (Looh et al., 2022; Díaz-Zúñiga et al., 2019).

*A. actinomycetemcomitans* is a genetically highly adaptable bacterium (Kittichotirat et al., 2016; Chen et al., 2020). This was first evidenced by serotyping, which revealed the existence of at least seven different serotypes (named a to g) that show variations in the O-antigen of their lipopolysaccharides (LPS) (Tsuzukibashi et al., 2014). The serotypes a, b and c are most commonly encountered. However, their prevalence varies with geographical locations and ethnicity. For instance, in the UK and Turkey, the serotype a is most common (Akrivopoulou et al., 2017; Doğan et al., 2016). In Morocco (Haubek et al., 2008), Spain (Mínguez et al., 2014), Germany (Kim et al., 2009) and India (Suprith et al., 2018) the serotype b is predominant. In many Asian countries, such as China (Mombelli et al., 1999), Japan (Wang et al., 2005; Yoshida et al., 2003), Thailand (Pahumunto et al., 2015) and Korea (Kim et al., 2009), as well as Brazil (Roman-Torres et al., 2010) and the USA (Chen et al., 2010), serotype c isolates are most frequent. Serotype variations are associated with differential clinical manifestations and the pathogenicity of *A. actinomycetemcomitans*. Isolates with the serotype b are often cultured from patients with severe periodontitis (Haubek et al., 2008; Cortelli et al., 2012), and their high virulence has been confirmed *in vitro* infection experiments (Monasterio et al., 2018; Díaz-Zúñiga et al., 2017).

**Fig. 1:**
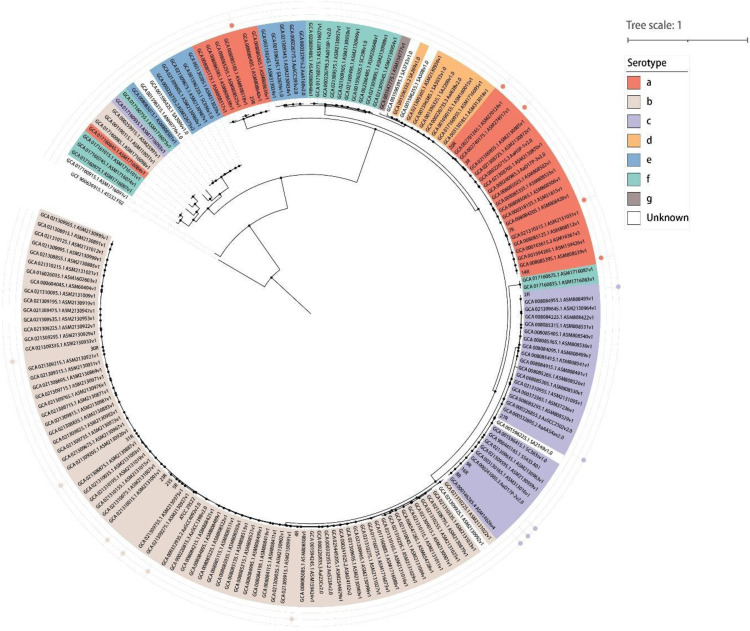
**Circular phylogenetic tree of *A. actinomycetemcomitans*.** The tree is based on marker genes of *A. actinomycetemcomitans* retrieved from the UniRef90 database. It was generated using PhyloPhlAn (v3.1.1) and visualized with iTOL (http://itol.embl.de/). Isolates characterized by integrated proteomics, virulence and immune evasion analyses are marked by color-coded bullets in the outer circles (Fu et al., 2023; Y. Fu et al., 2022).

In recent years, the phylogenetic divergence and genomic plasticity of *A. actinomycetemcomitans* isolates have become more clearly evident by (whole-genome) sequencing. This allows a distinction of five clades that overlap with the known serotypes: clade a/d includes isolates with serotypes a and d, clade b includes serotype b isolates, clade c includes serotype c isolates, clade e/f includes serotype e and f isolates, and clade e’ represents an outgroup of serotype e isolates (Kittichotirat et al., 2016; Kittichotirat et al., 2011). Furthermore, amongst isolates with serotypes a, b and c, three lineages were distinguished. Lineage I includes isolates related with serotypes b and c, lineage II includes the ‘common’ serotype a isolates, and lineage III includes ‘aberrant’ serotype a isolates (Nedergaard et al., 2019; Fu et al., 2023). Interestingly, isolates of lineage II are competent for genetic transformation (Nedergaard et al., 2019), suggesting that this mechanism for horizontal gene transfer (HGT) has contributed to the exchange and subsequent evolution of genomic islands in *A. actinomycetemcomitans*. In addition, prophages have been described for this bacterium, which implicate transduction by phages in HGT as well (Stevens et al., 2013). Genetic exchange by HGT probably led to the evolution of at least two serotype-specific genomic islands with distinct genes located at different loci in serotype a and serotype b-f isolates (Kittichotirat et al., 2022). The idea that phages have shaped the evolution of *A. actinomycetemcomitans* can also be inferred from the identification of genes for ‘clustered regularly interspaced short palindromic repeats’ (CRISPRs) and the expression of CRISPR-associated proteins in the common serotype a isolates (Fu et al., 2023). These CRISPRs and related proteins could be involved in phage immunity, but they may also contribute to virulence as was shown for *Campylobacter jejuni* (Saha et al., 2020). To visualize the evolutionary relationships among strains, a phylogenetic tree was constructed using marker genes of *A. actinomycetemcomitans* retrieved from the UniRef90 database, comprising a total of 186 strains. These included 29 serotype a strains, 85 serotype b strains, 29 serotype c strains, 8 serotype d strains, 11 serotype e strains, 16 serotype f strains, 1 serotype g strain, and 7 strains with unknown serotypes. *Aggregatibacter aphrophilus* ATCC 33,389 was used as the outgroup for tree rooting. As shown in Fig. 1, the majority of serotype b isolates formed a distinct cluster, with only three strains diverging from the main group. Most serotype c strains clustered closely and were positioned near the serotype b group, suggesting a close evolutionary relationship. Serotype a strains largely clustered with serotype d strains, indicating potential phylogenetic relatedness. A subset of serotype e strains grouped together with the majority of serotype f strains, forming a separate cluster.

## Leukotoxin A of *A. actinomycetemcomitans*

An important genomic island of *A. actinomycetemcomitans* carries the *ltxCABD* operon that encodes the major leukotoxin A (LtxA) and its associated secretion system. Also for this island, serotype- and clade-specific differences have been reported (Kittichotirat et al., 2022). In addition, a genetic variation within this island in serotype b isolates, referred to as the JP2 genotype, is related to severe periodontitis in young individuals (Haubek et al., 2008; Khzam et al., 2022). In particular, a deletion of 530 base pairs (bp) in the promotor region of the *ltxCABD* operon of JP2 isolates leads to overexpression of LtxA and enhanced leukotoxicity (Tsai et al., 2018). Genomic plasticity is also associated with elevated LtxA expression and progressive periodontitis observed in a Swedish cohort study, as evidenced for a subgroup of non-JP2 serotype b isolates of *A. actinomycetemcomitans* that carry the *cagE* gene (Johansson et al., 2017; Johansson et al., 2019). Moreover, two additional mutations in the *ltxCABD* promotor leading to higher LtxA production and leukotoxicity were recently recognized, involving deletions of 640 or 230 bp, respectively (Claesson et al., 2020; Claesson et al., 2015). Additional *ltxCABD* promotor mutations with varying effects on leukotoxicity were identified (Claesson et al., 2020; He et al., 1998), underpinning the view that such mutations can shape the virulence of *A. actinomycetemcomitans.* Here it is noteworthy that this type of mutations seem to occur quite frequently, as one study detected 33 *ltxCABD* promoter mutations amongst 693 *A. actinomycetemcomitans* isolates, with 30 of them belonging to the JP2 genotype (Claesson et al., 2020).

While LtxA is generally regarded as a major virulence factor of *A. actinomycetemcomitans*, the research documented in recent studies shows that it is certainly not the only important virulence factor. In particular, combined proteomics studies and infection experiments with strains belonging to serotypes a, b or c (marked in Fig. 1) show that certain isolates that produce relatively low levels of LtxA are apparently more virulent than other isolates that produce relatively high levels of LtxA (Fu et al., 2023). Nonetheless, previous studies have reported a compelling association between LtxA production and the clinical presentation of *A. actinomycetemcomitans*. Isolates from patients with aggressive periodontitis tended to produce high levels of LtxA, whereas isolates from healthy individuals tended to produce much lower LtxA levels (Haubek et al., 2008). Importantly, individuals carrying *A. actinomycetemcomitans* with the highly leukotoxic JP2 genotype showed a substantially increased odds ratio for clinical attachment loss and aggressive periodontitis compared to carriers of non-JP2 strains (Haubek et al., 2008; Mínguez et al., 2014). The markedly elevated odds ratio underscores the notion that specific *A. actinomycetemcomitans* genotypes/phenotypes, including high-level LtxA expression, are strongly associated with severe periodontal tissue destruction. In addition, high LtxA expression levels were also associated with the onset and progression of periodontitis (Khzam et al., 2022; Fine et al., 2019).

In initial studies, LtxA was reported to specifically target immune cells from humans or Old World primates, including neutrophils, macrophages and lymphocytes (Vega et al., 2019; Ozuna et al., 2021; Kelk et al., 2022). Neutrophils are the immune cells that form a first line of defense against invasive pathogens and, accordingly, they form the majority of leukocytes recruited into infected periodontal pockets (Jiang et al., 2021). Upon exposure to LtxA, neutrophils will release various granule components, including the neutrophil elastase (NE). This protease is present in elevated amounts in the saliva of periodontitis patients and eventually causes detachment and death of human gingival epithelial cells and human gingival fibroblasts (Hiyoshi et al., 2019). Moreover, LtxA provokes the release of neutrophil extracellular traps (NETs) (Hirschfeld et al., 2016), which induces periodontitis and further tissue destruction (Wang et al., 2021). Intriguingly, LtxA was shown to trigger hypercitrullination through the release of human peptidyl-arginine deiminases by the LtxA-affected neutrophils. These enzymes catalyze the conversion of positively charged arginine residues into neutral citrulline residues, a feature that has been implicated in the onset of RA which is characterized by high levels of auto-antibodies against citrullinated proteins (Konig et al., 2016). In macrophages, LtxA was shown to activate the ‘nucleotide-binding oligomerization domain, leucine-rich repeat and pyrin domain-containing 3′ (NLRP3) inflammasome, which can induce bone resorption and pro-inflammatory cell death (Kelk et al., 2022). However, the detrimental effects of LtxA are not limited to immune cells, since it also shows toxicity towards endothelial cells and erythrocytes at high concentrations (Looh et al., 2022; Krueger and Brown, 2020). These observations clearly associate LtxA with periodontitis, periodontal tissue destruction, and eventually systemic diseases, such as RA.

## Two distinct phenotypes of *A. actinomycetemcomitans*

**Fig. 2:**
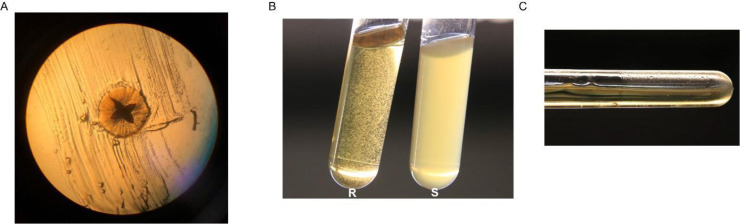
**Features of clinical *A. actinomycetemcomitans* isolates.** Clinical isolates of *A. actinomycetemcomitans* cultured on brain heart infusion (BHI) agar supplemented with 5 % l-cysteine, 5 mg/L hemin and 1 mg/L menadione and grown at 37 °C and 5 % CO_2_ for three days form small colonies that display a star shape upon scraping. The image in panel A was taken with a light microscope at 100x magnification. Upon 40 h culturing in BHI broth with the same supplementation, *A. actinomycetemcomitans* with the rough (R) colony phenotype presents granules in the broth (B) and biofilms on the walls of the tube (C). In contrast, *A. actinomycetemcomitans* with the smooth (S) colony phenotype forms homogeneous cultures in BHI broth (B).

As the genus name *Aggregatibacter* indicates, clinical *A. actinomycetemcomitans* isolates tend to aggregate, forming small granules in broth and biofilms on the walls of culture vessels (Fig. 2B,C). On solid media, the isolates form small rough colonies with a characteristic star-shaped and transparent appearance (Fig. 2A). However, after a few passages in the laboratory, the rough colony phenotype is lost and the bacteria form larger smooth colonies with an opaque appearance. The resulting strains with the smooth colony phenotype form homogeneous cultures in broth (Fig. 2B). Although essentially all clinical isolates of *A. actinomycetemcomitans* have the rough colony phenotype, a 2001 review referred to nine invasive clinical isolates with the smooth phenotype (Kachlany et al., 2001). However, this is certainly not the case for all invasive *A. actinomycetemcomitans* isolates, since two serotype c isolates that were recently collected from tissue of a sternum wound and thoracic pus at the University Medical Center Groningen, the Netherlands, presented the rough colony phenotype. Altogether, to the best of our knowledge, *A. actinomycetemcomitans* bacteria with the smooth colony phenotype have not been isolated from the oral cavity of patients with periodontitis. However, we cannot exclude the possibility that *A. actinomycetemcomitans* bacteria without fimbriae or with low-level fimbriation do occasionally emerge in the oral cavity.

**Fig. 3:**
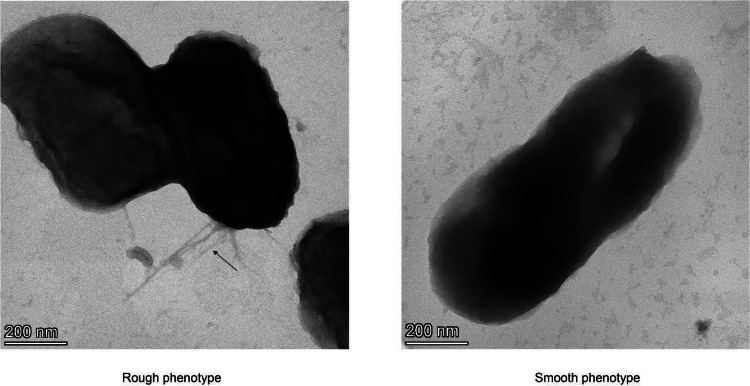
**Morphology of *A. actinomycetemcomitans* bacteria with the rough or smooth colony phenotypes.***A. actinomycetemcomitans* bacteria with the rough or smooth colony phenotypes were grown in BHI broth and subsequently imaged by transmission electron microscopy after negative staining with uranyl acetate. Size bars indicate the magnification. The arrow marks fimbriae of bacteria with the rough colony phenotype.

The rough colony phenotype is dependent on the expression of fimbriae with a diameter of 6 to 7 nm and variable length (Fu et al., 2022; Fine et al., 1999; Wang et al., 2005). As was shown by transmission electron microscopy (TEM) in Fig. 3, *A. actinomycetemcomitans* bacteria with the rough colony phenotype present bundles of long and thick fimbriae on their cell surface, while their smooth derivatives display short, thin or even no fimbriae. The fimbrial biogenesis in *A. actinomycetemcomitans* is controlled by the *flp* operon, which includes 14 genes (*flp-1-flp-2-tadV-rcpCAB-tadZABCDEFG*) (Danforth et al., 2019). Genome sequencing showed that smooth strains contain mutations in the *flp* promotor region, the repair of which leads to a restoration of the rough colony phenotype (Fu et al., 2022; Wang et al., 2005). Nine genes in the *flp* operon (*i.e.flp-1, rcpA, rcpB, tadB, tadD, tadE* and *tadF*) are indispensable for expression of fimbriae, while strains lacking the other five genes (*i.e.flp-2, tadZ, tadA, tadC* and *tadG*) display reduced fimbriation, or fimbriae with a different appearance (Perez et al., 2006; Wang and Chen, 2005). For one smooth strain, it was observed that the *flp-1* to *tadC* genes were lost during rough-to-smooth conversion (Fu et al., 2022). To date, only one study has reported reversion of the smooth to the rough colony phenotype upon culturing on a low-humidity solid medium (Pei et al., 2013). The respective reverted bacteria expressed fimbriae, but did not present star-shaped colonies. Unfortunately, the reversions were not identified by sequencing but, in case of point mutations in the *flp* promoter region, it is not surprising that reverted bacteria with the rough phenotype may appear. Notably, the rough to smooth conversion can be observed within six passages on BHI broth, which suggests that genetic instability is a general trait of *A. actinomycetemcomitans*, as can also be inferred from the rapid appearance of mutations in the *ltxCABD* promoter region.

Interestingly, *A. actinomycetemcomitans* bacteria with the smooth or rough colony phenotypes can differ not only in fimbrial expression and adhesion, but also in the localization and delivery of LtxA (Fu et al., 2022). *A. actinomycetemcomitans* isolates with the rough colony phenotype that expressed LtxA at high levels displayed LtxA retention to the cells, whereas derivative strains with the smooth colony phenotype released nearly all LtxA into their extracellular milieu (Fu et al., 2022). This suggests that highly fimbriated strains are more prone to retain LtxA in association with the bacterial outer membrane, which may have important consequences for virulence.

## Fimbriae are crucial for *A. actinomycetemcomitans*

Fimbrial appendages on the bacterial cell surface facilitate interactions between pathogens and the cells and tissues of their host. Such interactions are important for host colonization and the onset of infection (Reed and Williams, 1978; Sharma et al., 2021). In *A. actinomycetemcomitans* the fimbriae also facilitate auto-aggregation and the formation of biofilms, leading to persistent colonization of periodontal tissues and bacterial evasion of periodontal treatment with antimicrobial agents (Pourhajibagher et al., 2018). Furthermore, *flp-1* and *tadA* mutants, which lack fimbriae, failed to colonize the oral cavity of rats, did not elicit immune responses in rats, and did not cause bone loss in a murine infection model (Schreiner et al., 2003). This aligns well with the afore-mentioned notion that LtxA can be retained to the cells by fimbriae (Fu et al., 2022), thereby indirectly increasing the local toxin exposure at sites where the bacteria adhere to epithelial surfaces and immune cells. By enhancing colonization density and persistence within periodontal niches, fimbriae will thus potentiate leukocyte killing and immune evasion by LtxA. For another oral pathogen, *P. gingivalis*, the fimbrial adhesins are also known to be important for binding to other microbes, colonization of tissues, nutrient acquisition and invasion of immune cells and host tissues (Meghil et al., 2022). These observations together with recent findings showing reduced virulence of smooth *A. actinomycetemcomitans* strains compared to the respective parental isolates with the rough colony phenotype (Fu et al., 2022) imply that the disruption of fimbrial functions could be a very effective approach to prevent or treat periodontitis, and to prevent periodontitis-associated diseases that involve the bacterial translocation to non-oral body sites (Heidler et al., 2021).

## Outer membrane vesicles (OMVs)

OMVs are produced naturally by Gram-negative bacteria. Such vesicles are spheroid particles with a diameter of around 10–300 nm. The OMVs are pinched off from the cell envelope and, accordingly, they contain phospholipids, LPS and proteins. However, they also contain nucleic acids (Avila-Calderón et al., 2021).

OMVs have been reported to serve many different functions in nutrient acquisition, cell-cell communication, bacterial aggregation, biofilm formation, antibacterial activity, virulence, the evasion of host immune responses and antimicrobial resistance (Monteiro et al., 2025; Peregrino et al., 2024). In accordance with these functions, multiple OMV-associated virulence factors involved in immunoreactivity and proinflammatory activity, cytotoxicity, adhesion and invasion, immune evasion, drug targeting and the scavenging of iron and nutrients were identified by proteome analyses (Kieselbach et al., 2015; Fu et al., 2025). In particular, the OMVs of *A. actinomycetemcomitans* were proposed to act as vehicles for delivery of the virulence factors LtxA and cytolethal distending toxin (CDT) into human host cells (Rompikuntal et al., 2012; Nice et al., 2018). CDT is a genotoxic virulence factor that induces DNA damage-mediated cell-cycle arrest and apoptosis in epithelial and immune cells (Boesze-Battaglia et al., 2016). The DNase-like activity of the CdtB subunit disrupts host cell turnover and immune competence, which may provoke chronic inflammation and periodontal tissue breakdown (Boesze-Battaglia et al., 2016). CDT is therefore regarded as a factor that contributes to impaired host defense and disease persistence in the periodontal environment. Furthermore, OMVs of *A. actinomycetemcomitans* were shown to induce the ‘nucleotide-binding oligomerization domain-containing protein 1′ (NOD1)- and NOD2-dependent NF-κB activation, which has been implicated in alveolar bone resorption (Thay et al., 2014). OMVs from *A. actinomycetemcomitans* were also shown to provide serum protection, allowing the bacteria to translocate from the oral cavity to other body sites (Lindholm et al., 2020).

OMVs from *A. actinomycetemcomitans* can be internalized by non-phagocytic host cells, such as HeLa cells and human gingival fibroblasts, via multiple pathways including clathrin-mediated endocytosis and fusion with plasma membranes in a cholesterol-dependent process (Rompikuntal et al., 2012; Thay et al., 2014). In this context, it is noteworthy that the internalization of OMVs from *A. actinomycetemcomitans* into host cells was LtxA- or CDT-independent (Rompikuntal et al., 2012; Demuth et al., 2003). Furthermore, the LPS from *A. actinomycetemcomitans* has been implicated in the binding of interleukin-8, which may allow OMVs to influence the migration of neutrophils (Ahlstrand et al., 2019). Other OMV cargo from *A. actinomycetemcomitans* that may influence the behavior of host cells are small RNA molecules of microRNA size (msRNAs) that can modulate the host immune response (Ha et al., 2020; Choi et al., 2017; Han et al., 2019). Accordingly, such extracellular RNAs (exRNAs) may be regarded as biomarkers for systemic diseases (Lee, 2020). Interestingly, OMVs from *A. actinomycetemcomitans* were, for instance, shown to cross the blood-brain barrier (BBB) after intracardiac injection and exRNAs contained in these OMVs were implicated in the activation of TNF-α in the mouse brain cortex (Han et al., 2019). Furthermore, OMV-associated exRNAs were delivered into meningeal macrophages and cortex microglial cells in a time-sequential manner upon tail-vein injection in a murine infection model, and such exRNAs induced the release of proinflammatory cytokine IL-6 in a murine microglial cell line (Ha et al., 2020). Altogether, these findings underpin the view that *A. actinomycetemcomitans* is not only involved in periodontitis, but that this bacterium may also act as a causative agent in systemic diseases through the strategic transfer of many different virulence factors, including proteins, LPS and RNA, over long distances with the help of OMVs.

## Fimbriae block the excretion of extracellular cytoplasmic proteins (ECPs)

Consistent with the important role of OMVs in the virulence of *A. actinomycetemcomitans*, the proteins that the bacteria release into their extracellular milieu, collectively referred to as the exoproteome, should be regarded as an important reservoir of virulence factors. This notion inspired extensive exoproteome analyses by mass spectrometry (MS). Interestingly, the MS analyses did not only uncover typical secretory proteins, or periplasmic and (outer) membrane proteins that can be associated with OMVs, but also many cytoplasmic proteins. Such extracellular cytoplasmic proteins (ECPs) may serve dual ‘moonlighting’ functions inside and outside the bacterial cell, for instance in the adhesion to host cells and tissues or detoxification of the environment (Ebner and Götz, 2019). However, ECPs may also be regarded as a sign of bacterial lysis. Interestingly, *A. actinomycetemcomitans* bacteria with the smooth colony phenotype excreted substantially more extracellular cytoplasmic proteins than bacteria with the rough colony phenotype. The simplest explanation for this observation is that bacteria with the smooth phenotype are more prone to lysis due to their reduced fimbriation. This would suggest that fimbriae serve an important role in maintaining the bacterial integrity, in addition to their adhesive functions. Conversely, the excretion of ECPs that could also promote adhesion to cells and tissues of the host might reflect a bacterial strategy to compensate, at least to some extent, for the absence of fimbriae. In any case, the enhanced release of ECPs by smooth colony variants of *A. actinomycetemcomitans* could not be associated with the other known mechanisms implicated in this process, such as (pro)phage activity or the secretion of cell envelope-destabilizing autolysins or toxins (Ebner and Götz, 2019; Ebner et al., 2017; Zhao et al., 2019; Schlag et al., 2010). The view that fimbriae counteract the excretion of ECPs is supported by the observation that *A. actinomycetemcomitans* isolates with the serotype a excreted significantly more ECPs than isolates with the serotypes b or c, whereas fewer fimbriae-associated proteins were identified for isolates with the serotype a (Fu et al., 2023). Interestingly, OMVs of *A. actinomycetemcomitans* carry also substantial amounts of ECPs, whereas bacteria with the smooth colony phenotype excrete substantially more OMVs than bacteria with the rough phenotype. Thus, there seems to be an intimate relationship between the release of OMVs and ECPs, which suggests that cell envelope destabilization is a main cause of ECP excretion in *A. actinomycetemcomitans*.

The high-level excretion of ECPs by non-fimbriated strains is not an exclusive trait of *A. actinomycetemcomitans*, since it is observed in at least one other oral pathogen, namely *P. gingivalis* (Stobernack et al., 2016). *P. gingivalis* expresses long and short fimbriae, which are also known as the major and minor fimbriae, respectively (Meghil et al., 2022). The major fimbrial subunit protein type-1 (FimA) was not identified in the reference strains W83 and MDS45, which present higher numbers of identified ECPs than the fimbriated strain ATCC 33,277 expressing FimA (Stobernack et al., 2016).

Despite the higher amounts of ECPs excreted by *A. actinomycetemcomitans* with the smooth colony phenotype, which could be regarded as a sign of bacterial lysis, these smooth strains grow much better in broth than the parental strains with the rough colony phenotype (Fig. 2). This implies that the smooth strains are fitter for culturing under laboratory conditions. Likewise, it has been reported that an *Escherichia coli*strain lacking fimbriae grew better compared with its parental strain, and that gene expression was generally upregulated in the non-fimbriated mutant (Qiao et al., 2021). This is in accordance with the fact that the synthesis and assembly of fimbriae require energy that cannot be used for other processes. Furthermore, fimbriae may alter the physiochemical properties of the bacterial cell surface. These ideas are fully in line with the different phenotypes of *A. actinomycetemcomitans* with the rough and smooth colony phenotypes (Fig. 1). Likewise, assembly of the so-called CupD fimbriae on the surface of *Pseudomonas aeruginosa* was shown to result in a small colony morphotype, increased biofilm formation and decreased motility (Mikkelsen et al., 2009).

Lastly, while more ECPs were produced by *A. actinomycetemcomitans* strains with the smooth phenotype, isolates with the rough colony phenotype produced more extracellular virulence factors (Fu et al., 2023; Fu et al., 2022). This is in accordance with the infectious behavior of the rough isolates in humans, whereas the isolation of smooth strains from humans has not been verifiably documented. Based on these observations, it can be concluded that fimbriae are the key virulence factors of *A. actinomycetemcomitans*, a view that is fully supported by the correlation of the exoproteome data and *in vitro* virulence assays with human neutrophils and gingival epithelial cells (Fu et al., 2023; Fu et al., 2022). In this context it is important to note that, while ECP excretion is a prominent feature of *A. actinomycetemcomitans* bacteria with the smooth colony phenotype, this does not mean that bacteria with the rough colony phenotype do not release ECPs - they merely release lower amounts of ECPs. Therefore, even though the smooth colony phenotype of *A. actinomycetemcomitans* is essentially observed under *in vitro* laboratory conditions, experimental comparisons between smooth and rough colony variants provide important mechanistic insights into virulence regulation and the potential contribution of ECPs to virulence. Clearly, the rough and smooth colony phenotypes reflect distinct regulatory and structural states that influence the bacterial surface architecture, extracellular protein secretion and outer membrane properties. Differences in ECP release between smooth and rough strains therefore illuminate how alterations in the bacterial envelope modulate the localization and delivery of virulence factors, such as LtxA. Thus, it seems that fimbriae influence the bacterial virulence not only by mediating adhesion, but also by modulating the spatial distribution and functional impact of ECPs and OMVs. Potentially, fimbrial structures may facilitate the local retention of secreted virulence factors at the bacterial surface, thereby creating a protective microenvironment that limits immune clearance, while also enabling concentrated delivery of OMV-associated toxins, such as LtxA and CDT, to host cells either in close proximity or at sites distant from the bacterium.

## Concluding remarks and future perspectives – fimbriae as druggable targets

Altogether, recent studies provide a deeper understanding of the contributions of extracellular proteins to the morphological features, growth behavior and virulence of different *A. actinomycetemcomitans* isolates belonging to the globally predominant serotypes a, b and c (Fu et al., 2023; Fu et al., 2022). The results also show how OMVs of *A. actinomycetemcomitans* interact with human neutrophils, provoking them to prematurely discharge their antimicrobial armory represented by NETs and antimicrobial proteins (Fu et al., 2025). It thus seems that OMVs serve an important immune evasive function for *A. actinomycetemcomitans* in the oral cavity. Importantly, the correlation of identified virulence factors and the virulence of *A. actinomycetemcomitans* in different *in vitro* infection models highlights the key virulence factors of this pathogen, especially the fimbriae-associated proteins. Since fimbriae are primarily known to serve attachment functions, it seems plausible that they are needed to bring the bacteria and cells of the host in close proximity, such that virulence factors like LtxA and CDT can execute their cytotoxic effects. Possibly, the fimbrial proteins included in OMVs also facilitate association of these bacterial satellites with host cells, but this remains to be shown in future studies. In any case, the observations presented in this review suggest that the bacterial fimbriae are prime targets for preventive and therapeutic interventions against the pathogen *A. actinomycetemcomitans.* In particular, disruption of the interactions between the pili and the host cells using small molecule drugs or immunization approaches could provide added value by complementing the present-day treatments based on periodontal care and surgical interventions.

Vaccination is one of the most effective strategies to control and reduce the mortality and morbidity caused by infectious diseases (Cid and Bolívar, 2021). The conventional approaches include live-attenuated, inactivated, subunit and toxoid vaccines that generally target proteins and capsule polysaccharides on the bacterial cell surface (Wadhwa et al., 2020). On the other hand, novel more sophisticated vaccines involve the delivery of DNA or RNA to the host, such that the host will produce relevant antigens of the pathogen (Wadhwa et al., 2020). As can be concluded from the major success in fighting the recent Covid-19 pandemic, messenger RNA (mRNA)-based vaccines are highly attractive for addressing infectious diseases due to their efficacy, safety and ease of delivery (Deng et al., 2022). Hence, it is an attractive idea to develop such vaccines also for the treatment of bacterial infections, including recurring periodontal disease. Fimbrial subunits and particular toxins of *A. actinomycetemcomitans* appear to be attractive targets for such vaccines. Fimbriae have been previously explored as vaccine antigens in different bacterial pathogens, where anti-fimbrial immunity can block adhesion and colonization (Gorringe and Vaughan, 2014). The most prominent example in human medicine is the acellular pertussis vaccine, in which the fimbrial antigens Fim2 and Fim3 contribute to protection by limiting respiratory tract colonization by *Bordetella pertussis* (Alexander et al., 2012; Hallander et al., 2014). In parallel, fimbriae-based vaccines have long been established as the gold standard in veterinary medicine for the prevention of enteric *E. coli*infections in livestock, providing compelling real-world evidence that neutralization of these surface adhesion structures effectively prevents severe diarrheal disease (Broeck et al., 1999).

Despite the successes, the broader application of fimbriae-based vaccines has been constrained by antigenic and phase variation, whereby bacteria alter fimbrial expression or structure to evade host immunity (Tajuelo et al., 2024). This limitation will necessitate the targeting of conserved adhesive elements rather than entire fimbriae. This view is supported by studies on vaccines that specifically target fimbrial tip-associated adhesins, such as the FimH-containing FimCH complex. In this case, clinical follow-up studies reported up to 75 % reduction in recurrent urinary tract infections caused by antibiotic resistant Enterobacteriaceae (Perer et al., 2024). Furthermore, another study indicated that immunization of mice with the FimA and MrkA subunits from fimbriae of *Klebsiella pneumoniae* significantly reduced the severity of infections in a pneumonia model (Assoni et al., 2025).

Following up on this line of reasoning, a combined vaccine targeting fimbriae and LtxA of *A. actinomycetemcomitans* could simultaneously inhibit adhesion of the bacteria to the human tissues and neutralize a major toxin. Such a multicomponent strategy may provide broader protection than single-antigen vaccines. Inhibiting the virulence of *A. actinomycetemcomitans* bacteria with a competitive harmless non-virulent *A. actinomycetemcomitans* strain would also be worth investigating but, in this case, it would be necessary that the bacteria can still adhere to the periodontal tissues, which requires the expression of fimbriae. It is presently uncertain whether such a non-virulent strain that is completely safe for application in humans can be developed and we anticipate regulatory hurdles for administrating non-virulent *A. actinomycetemcomitans* to patients. In that sense, a more promising strategy might be the identification of probiotic bacteria that can outcompete *A. actinomycetemcomitans* in the oral cavity.

Vaccination against *A. actinomycetemcomitans* and *P. gingivalis* could also be beneficial for the prevention of systemic diseases associated with periodontitis. This view is supported by a cohort study, which showed that patients with early and established RA presented increased levels of anti-LtxA IgM compared with matched non-RA controls and periodontally healthy individuals. The anti-LtxA IgM levels were associated with early-stage RA, suggesting a link between *A. actinomycetemcomitans* and systemic diseases (Gomez-Bañuelos et al., 2020). However, before vaccination approaches against *A. actinomycetemcomitans* can be developed, it will be important to determine carefully whether they are safe and which individuals will benefit the most from them. Clearly, since periodontal disease is one of the most wide-spread diseases at the global scale, with people in developing countries being most severely affected, the development of alternative treatments based on relatively cheap antimicrobial drugs or disinfectants should also be considered next to vaccine development.

## Declaration of competing interest

The authors declare the following financial interests/personal relationships which may be considered as potential competing interests:

Yanyan Fu reports financial support was provided by the China Scholarship Council. Qiqi Pan reports financial support was provided by the China Scholarship Council. If there are other authors, they declare that they have no known competing financial interests or personal relationships that could have appeared to influence the work reported in this paper.
